# Systematic Review on Pathophysiological Complications in Severe COVID-19 among the Non-Vaccinated and Vaccinated Population

**DOI:** 10.3390/vaccines10070985

**Published:** 2022-06-21

**Authors:** Ali A. Rabaan, Muhammed A. Bakhrebah, Abbas Al Mutair, Saad Alhumaid, Jumana M. Al-Jishi, Jehad AlSihati, Hawra Albayat, Ahmed Alsheheri, Mohammed Aljeldah, Mohammed Garout, Wadha A. Alfouzan, Yousef N. Alhashem, Salma AlBahrani, Saleh A. Alshamrani, Sultan Alotaibi, Abdullah A. AlRamadhan, Hanadi N. Albasha, Khalid Hajissa, Mohamad-Hani Temsah

**Affiliations:** 1 Molecular Diagnostic Laboratory, Johns Hopkins Aramco Healthcare, Dhahran 31311, Saudi Arabia; 2 College of Medicine, Alfaisal University, Riyadh 11533, Saudi Arabia; 3 Department of Public Health and Nutrition, The University of Haripur, Haripur 22610, Pakistan; 4 Life Science and Environment Research Institute, King Abdulaziz City for Science and Technology (KACST), Riyadh 11442, Saudi Arabia; mbakhrbh@kacst.edu.sa; 5 Research Center, Almoosa Specialist Hospital, Al-Ahsa 36342, Saudi Arabia; abbas.almutair@almoosahospital.com.sa; 6 College of Nursing, Princess Norah Bint Abdulrahman University, Riyadh 11564, Saudi Arabia; 7 School of Nursing, Wollongong University, Wollongong, NSW 2522, Australia; 8 Nursing Department, Prince Sultan Military College of Health Sciences, Dhahran 33048, Saudi Arabia; 9 Administration of Pharmaceutical Care, Al-Ahsa Health Cluster, Ministry of Health, Al-Ahsa 31982, Saudi Arabia; saalhumaid@moh.gov.sa; 10 Internal Medicine Department, Qatif Central Hospital, Qatif 32654, Saudi Arabia; dr.jmsj@gmail.com; 11 Internal Medicine Department, Gastroenterology Section, King Fahad Specialist Hospital, Dammam 31311, Saudi Arabia; jehadalsaihati@gmail.com; 12 Infectious Disease Department, King Saud Medical City, Riyadh 7790, Saudi Arabia; hhalbayat@gmail.com (H.A.); drahmedajlan@hotmail.com (A.A.); 13 Department of Clinical Laboratory Sciences, College of Applied Medical Sciences, University of Hafr Al Batin, Hafr Al Batin 39524, Saudi Arabia; mmaljeldah@uhb.edu.sa; 14 Department of Community Medicine and Health Care for Pilgrims, Faculty of Medicine, Umm Al-Qura University, Makkah 21955, Saudi Arabia; magarout@uqu.edu.sa; 15 Department of Microbiology, Faculty of Medicine, Kuwait University, Safat 13110, Kuwait; alfouzan.w@hsc.edu.kw; 16 Microbiology Unit, Department of Laboratories, Farwania Hospital, Farwania 85000, Kuwait; 17 Department of Clinical Laboratory Sciences, Mohammed AlMana College of Health Sciences, Dammam 34222, Saudi Arabia; yousefa@machs.edu.sa; 18 Infectious Disease Unit, Specialty Internal Medicine, King Fahd Military Medical Complex, Dhahran 31932, Saudi Arabia; drsalma1@hotmail.com; 19 Department of Clinical Laboratory Sciences, College of Applied Medical Sciences, Najran University, Najran 61441, Saudi Arabia; saalshamrani@nu.edu.sa; 20 Molecular Microbiology Department, King Fahad Medical City, Riyadh 11525, Saudi Arabia; salotaibi1@gmail.com; 21 Laboratory and Toxicology Department, Security Forces Specialized Comprehensive Clinics, Al-Ahsa 36441, Saudi Arabia; aramadhan@moimsd.gov.sa; 22 Department of Infection Prevention and Control, Obeid Specialized Hospital, Riyadh 12627, Saudi Arabia; drhanadi@hotmail.com; 23 Department of Medical Microbiology & Parasitology, School of Medical Sciences, Universiti Sains Malaysia, Kubang Kerian 16150, Malaysia; khalid541983@yahoo.com; 24 Pediatric Department, College of Medicine, King Saud University, Riyadh 11451, Saudi Arabia; mtemsah@ksu.edu.sa

**Keywords:** SARS-CoV-2, COVID-19, multiorgan failure, vaccination, multisystem inflammatory syndrome, ACE2 receptor

## Abstract

COVID-19, caused by SARS-CoV-2, is one of the longest viral pandemics in the history of mankind, which have caused millions of deaths globally and induced severe deformities in the survivals. For instance, fibrosis and cavities in the infected lungs of COVID-19 are some of the complications observed in infected patients post COVID-19 recovery. These health abnormalities, including is multiple organ failure—the most striking pathological features of COVID-19—have been linked with diverse distribution of ACE2 receptor. Additionally, several health complications reports were reported after administration of COVID-19 vaccines in healthy individuals, but clinical or molecular pathways causing such complications are not yet studied in detail. Thus, the present systematic review established the comparison of health complication noted in vaccinated and non-vaccinated individuals (COVID-19 infected patients) to identify the association between vaccination and the multiorgan failure based on the data obtained from case studies, research articles, clinical trials/Cohort based studies and review articles published between 2020–2022. This review also includes the biological rationale behind the COVID-19 infection and its subsequent symptoms and effects including multiorgan failure. In addition, multisystem inflammatory syndrome (MIS) has been informed in individuals post vaccination that resulted in multiorgan failure but, no direct correlation of vaccination with MIS has been established. Similarly, hemophagocytic lymphohistiocytosis (HLH) also noted to cause multiorgan failure in some individuals following full vaccination. Furthermore, severe complications were recorded in elderly patients (+40 years of age), indicates that older age individuals are higher risk by COVID-19 and post vaccination, but available literature is not sufficient to comply with any conclusive statements on relationship between vaccination and multiorgan failure.

## 1. Introduction 

**Figure 1:**
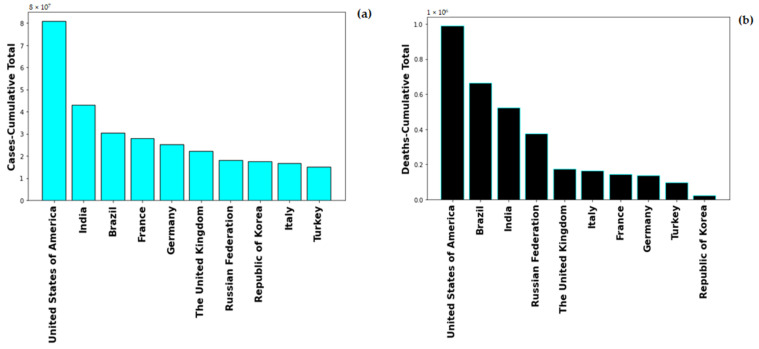
Total Number of (**a**) cases and (**b**) deaths for top 10 countries globally.

Global healthcare system was severely alarmed by the rate of Coronavirus-19 infection, on 1st May 2022, the global number of the novel Coronavirus-19 (nCOVID-19) infected patients stands at 513,348,944 with number of deaths at 6,260,762. The highest number of COVID-19 cases are in USA with 83,066,907 and also maximum number of COVID-19 induced deaths, i.e., 1,030,833. Figure 1 shows the cumulative cases and deaths for top 10 countries globally. United States of America has maximum number reported both in number of cases and number of deaths. The pandemic spread in multiple were lethal for numerous people and rendering others with severe deformities. The severity of COVID-19 enhances with the presence of co-morbidity factors such as diabetes, hypertension, immunocompromised conditions. 

**Figure 2:**
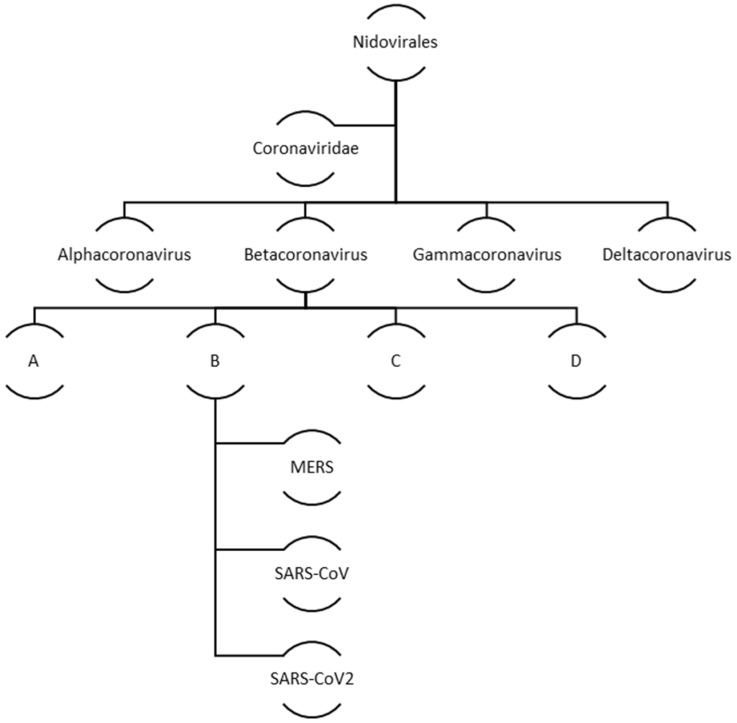
Evolution and classification of human coronavirus.

Coronaviruses belong to a big family of viruses, Coronaviridae that comprises of enveloped single stranded RNA viruses. They can infect human, bats, pangolin and other mammals, birds, livestock and even pets. There are four genera within the Coronaviridae family: α-Coronaviruses, β-Coronaviruses, γ-Coronaviruses, and δ-Coronaviruses. While the first two genera exclusively infect mammals the latter two have a diverse range of host species that also include birds. In case of humans and other animals, coronaviruses infections mainly manifest as the respiratory and enteric diseases. Figure 2 illustrates the family and evolution of corona virus. 

Most of the COVID-19 patients are mainly asymptomatic or display mild symptoms. The older age group and especially those suffering from other forms of illnesses, such as lung or heart diseases, are at greater possibility of developing the acute form of COVID-19 infections. The severe forms of the disease can also be observed in healthy younger individuals lacking any health disorder. In case of children, most of the COVID-19 patients either does not display any symptoms or show symptoms of milder magnitude. However, recent studies suggest the development of multisystem inflammatory syndrome thus significantly increasing the mortality rates [1,2,3]. The major symptoms displayed by the patients infected by SARS-CoV-2 are fever, dry cough, fatigue, dyspnea [4]. The cells of many organs possess the ACE2 receptor beyond which SARS-CoV-2 may spread within the host body causing multiple organ failure [5].

Although, various vaccine candidates have been approved by WHO and used in COVID-19 patients, the occurrence of multi-organ failure have still been reported by several case studies. This review discusses extensively, the co-relation between vaccination status of COVID-19 patients and occurrences of multi organ failure, which has not been covered elsewhere. This co-relation study shall help the researchers to study the long-term effects of COVID-19 vaccines on the virus infected and non-infected individuals. 

## 2. Methods

**Figure 3:**
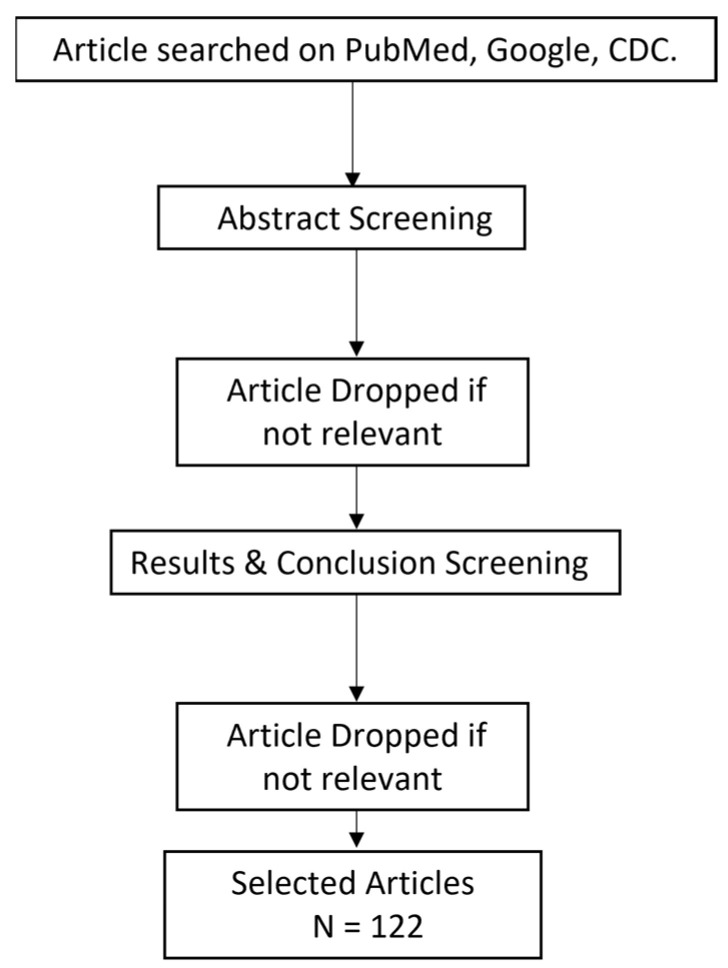
Flowchart for the selection of articles.

This metareview includes the data obtained from 38 research articles, 16 review articles, 32 case studies and 46 clinical trials/cohort-based studies conducted between 2020–2022 in various countries. Flowchart of articles selected is shown in Figure 3. 

The number of patients from cohort-based studies which displayed multi organ complications from vaccinated and non-vaccinated groups were used for further evaluations to draw the correlations. Official WHO web site https://covid19.who.int/ (accessed on 25 April 2022) was used for data collection on number of cases and vaccination. ‘R’ statistical package was used for the data analysis and plotting bar and stem plots. Images were drawn using power point Microsoft application and license free images web sources (https://www.pngwing.com/ (accessed on 25 April 2022)). Central disease control and prevention database is used to search for the case studies at https://wwwnc.cdc.gov/ (accessed on 25 April 2022) URL. Data collection in this study used following inclusion and exclusion criteria. 

Inclusion Criteria: Original research work, review on post COVID and post vaccination, Case series, Case studies, cross-sectional studies

Exclusion Criteria: News articles, un-authorized reports, and web articles.

## 3. Viral Pathogenesis 

### 3.1. Genomic Architecture 

Coronaviruses are named so due to their crownlike exterior owing to the occurrence of spike shaped glycoproteins on the viral envelope and belongs to the family of B—Coronaviruses [6]. The genome of COVID-19 is made up of single strand RNA, nearly 30 kb in length (sense strand) [7]. Two dominant open reading frames (ORFs) namely 1a and 1b constitutes approximately 70% of the viral genome. These ORFs codes for numerous conserved protein sequences with distinct functions such as replication of virus and evasion of host innate immune responses [8,9]. In the midst of all the coronavirus genome sequences, the genome of COVID-19 shares maximum similarity with two other coronaviruses, the bat coronavirus (96%) and the pangolin coronavirus (91%) [10,11]. 

### 3.2. Structural Elements 

**Figure 4:**
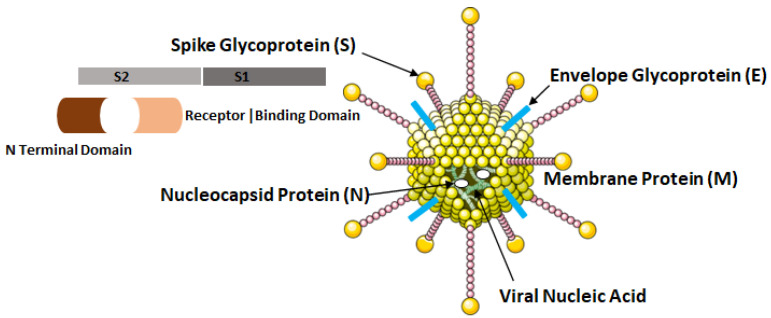
Structural elements of COVID-19 virus, i.e., SARS-CoV-2.

The COVID-19 virus possesses four major structural proteins, namely the spike glycoproteins, matrix glycoproteins, envelope glycoproteins and the nucleocapsid proteins as shown in Figure 4. All of these proteins can induce the immune response inside the hosts [12].

The nucleocapsid proteins are phosphorylated that enables them to bind to viral nucleic acid and carry out essential functions during the life cycle of the virus. The glycoprotein on viral envelope is a membrane integral protein which makes up the significant part of the viral structure. These proteins play essential role during assembly of the viral particles by cross linking with other structural components of the virus [13]. The viral spike glycoprotein spans the host membrane and is present in the outermost layer of the virus. The two subunits, S1 and S2 which makes up these proteins are processed by the host proteases. The S1 subunit consisting of two domains: (a) N terminal and (b) Receptor Binding Domain (RBD) influences the cellular tropism of the virus. After the fusion of viral envelope with host membrane, the S1 subunit is dissociated that induces a change in the conformation of S2 subunit which then act as an anchor for the viral membrane [8]. 

### 3.3. Host Cell Entry 

The SARS-CoV-2 virus enter the host cells using ACE2 (Angiotensin Converting Enzyme-2) which is a carboxypeptidase and maintains the balance of fluid and electrolytes essential for the proper functioning of multiple organs in the body. The binding affinity of ACE2 and the spike protein influences the severity and the outcome of COVID-19 infection. ACE-2 converts angiotensin-I (ang-I) to angiotensin-II (ang-II) which in turn can interact with the Angiotensin Type-1 Receptor to induce pro-inflammatory and fibrotic response in the host body [14,15]. The same ACE-2 is also involved in the conversion of ang-II to ang-I and further to ang. The Ang-I interacts with Mas receptors and reverses the downstream effects induced by ang-II. However, the affinity of ACE2 for ang-I is the highest, thus, favoring its conversion to ang-II [16]. In addition to ACE2, other receptors involved in the cellular entry of coronaviruses are Human Aminopeptidase N (APN) for Human Coronavirus 229E and Dipeptidyl Peptidase 4 in case of Middle East Respiratory Syndrome Related Coronaviruses [17].

### 3.4. Mechanism of Viral Replication 

The RBD domain of COVID-19 binds to ACE2 to enter into the host cells. This process is aided by the host cell surface TMPRSS2 (Transmembrane Serine Protease 2), cathepsin L and cathepsin B [18,19,20]. These interactions then induce the uptake of ACE2 receptor along with COVID-19 virus through the process of endocytosis that further led to the combination of the viral membrane with host cell. The intracellular COVID-19 then reduces the levels of ACE2 both at the mRNA and protein levels [21]. Inside the endosomes, the spike protein of the virus becomes cleaved by the cysteine proteases causing its cleavage to dissociate S1 subunit initially and then to expose the fusion peptide within the S2 subunit [21]. This event releases the packaged virus into cell cytoplasm to produce more progeny virions using the host cellular machinery. The viral genome consisting of a single stranded copy of RNA also act as a messenger RNA to direct the host ribosomes towards the synthesis of viral proteins. The naked RNA genome of the virus codes for polypeptides 1a and 1b which are then cleaved to form the non-structural proteins. These polypeptides then interact with viral genome to form the sub genomic viral particles that translate structural proteins [22]. Gordon et al., detected 332 protein–protein interactions (high confidence) between the COVID-19 and humans which were involved in virus life cycle [23]. Upon the production of all the viral proteins and assembled in the ER-Golgi compartments, the progeny virions are released through the process of exocytosis outside the host cell [22,24]. 

## 4. COVID-19 Induced Pathophysiological Complications

Pathophysiological changes or complications refer to altered physiological changes occurring in diseased state and its impact on the mortality and disease outcome. COVID-19 has been shown to affect multiple organs and systems in human body such as respiratory, cardiovascular, hematological, gastrointestinal, urogenital and central nervous system. This multi organ involvement and damage is due to abundance of ACE2 surface protein, a major route of virus entry on the cells of these organs/systems. These pathophysiological alterations involving multiple organs and systems are enlisted below. 

### 4.1. Multiple Organ Injury

Even though many aspects of SARS-CoV-2 virus-host interactions are unclear, some of them are shown to mimic the pathogenesis of other human coronaviruses. Five primary pathogenic processes underpin COVID-19 genesis and progression are: cytotoxicity induced by the virus directly into ACE2-expressing cells, RAAS dysregulation as a byproduct of virus-facilitated ACE2 downregulation, immunological dysregulation, endothelial cell damage and thrombosis, and tissue fibrosis [25]. The lungs have various characteristics that make them ideal for serving as a preliminary reservoir for viral transmission and replication from person to person. Due to high surface area of lung, it is more vulnerable to viruses absorbed through the air. It’s also well-vascularized, allowing virus particles to spread quickly to nearby organs. Additionally, ACE2 is expressed in a variety of lung cell types with alveolar epithelial type II cells displaying the highest abundance [26,27]. Various viral functional genes, such as those involved in the genome replication are expressed by alveolar epithelial type II cells, according to gene ontology enrichment study [27]. The generation and exocytosis releasing new viral progeny can cause pyroptosis. The host cell responds to the viral infection through the recognition of damage-associated molecular pattern (DAMP) signals. DAMP are also identified by nearby epithelial as well as endothelial cells in lung tissue, which further activates the transcription factors, interferon regulatory factor 3 (IRF3) and nuclear factor-B (NF-B), causing production and secretion of pro-inflammatory mediators [6]. Concurrently, type I interferons are secreted, which have antiviral effects through a variety of pathways. Antigen-presenting cells (APCs), such as resident tissue macrophages and dendritic cells, identify these first inflammatory signals and deliver the antigen to T_H_ cells, which subsequently prime other T_H_ cells, T_C_ cells, and B-cells in an immunocompetent reaction [28]. B-cells generate neutralizing antibodies against the spike protein and nucleocapsid, whereas T_C_ are cytotoxic and destroy host cells infected with virus immediately. Phagocytes eliminate apoptotic cells and viruses that have been neutralized, results in a well-coordinated infection elimination with little lung tissue harm [6,28]. A defective immune response, on the other hand, leads to increased inflammation, ineffective viral removal, hyperactivation of innate immune system and increased production of pro-inflammatory cytokines such as interleukin (IL)-1, IL-6, tumor necrosis factor (TNF), etc. The alveolar-capillary membrane is disrupted by this unmanaged inflammatory process, and viruses with the subsequent cytokine storm, can subsequently move to other body sites, leading to multi-organ failure [29]. In patients with acute COVID-19, histological investigation of lung cells revealed neutrophil migration into the alveolar space as well as acute capillaritis [30,31,32]. The initiation of the coagulation system, neutrophil extracellular traps (NETs), and leakage of tissue fluid in the sub-endothelial region may all be linked to the inflammatory reaction caused by the host-virus interaction in severe COVID-19 patients [30,33,34,35]. Individual heterogeneity in expression of ACE2, TMPRSS2 receptors, as well as genetic elements linked to inflammatory and immunological responses, might explain COVID-19’s diverse clinical symptoms [36,37,38,39]. As a result, patients with severe COVID-19 may experience increasing acute inflammatory reactions, which can lead to MODS and mortality [40,41]. The acute inflammatory responses leads to cytokine storm with significantly elevated levels of proinflammatory mediators, such as IL-1β, IL-2, IL-6, IL-7, IL8, TNF-α, interferon induced protein 10, monocyte chemoattractant protein 1, granulocyte colony-stimulating factor, macrophage inflammatory protein-1, platelet-derived growth factor, vascular endothelial growth factor [29,42,43,44,45]. This storm is pronounced in patients hospitalized to the intensive care unit, revealed by higher blood concentrations of various pro-inflammatory mediators, suggesting a link between illness severity and serum concentrations [29,43,44,45]. In acute COVID-19, abnormal CD4^+^ T_H_ cells co-expressing IFN- and G-CFS are also observed [46]. A higher proportion of neutrophils over lymphocytes ratio is linked to death in the hospitalized COVID-19 patients [47]. Patients with severe infection, however, may develop lymphopenia, which is defined by a large decrease in peripheral CD4^+^ as well as CD8^+^ T-cells. These counterbalancing responses could hinder SARS-CoV-2 from being completely cleared and may lead to the development of secondary infections likely to result in a sepsis-like condition [43,45,48].

Recently, a cohort-based study was conducted to assess the multi organ health in 443 non hospitalized German individuals with a previous mild to moderate COVID-19 infection compared to healthy uninfected controls. The assessment of pulmonary, cardiovascular, urogenital, and neurological systems was carried out, and the outcome was examined carefully. The assessment of cardiac system revealed enhanced levels of cardiac biomarkers along with lowered ventricular function. Ultrasonography revealed the presence of deep vein thrombosis indicated by the less compressible nature of femur veins. In addition, lower glomerular filtration rate and the increased mean thickness of brain cortex was observed. Other parameters such as brain volume and cognitive functions were non-impaired [49].

### 4.2. Gastrointestinal System

COVID-19 infected women are reported to illicit a stronger T-cell response than males, which may lead to more effective viral clearance [50]. The presence of stomach discomfort, nausea, diarrhea, and vomiting in a significant number of COVID-19 patients indicates virus induced gastrointestinal dysfunction [41,43,48,51,52]. ACE2 is abundant on the luminal surface of intestinal epithelial cells and functions as a co-receptor towards the amino acid absorption [26,53]. The nucleocapsid protein of COVID-19 is detected in various gastrointestinal regions [54,55] as well as in stool samples [56,57,58], suggesting that oral-fecal transmission is possible. Histopathological study of COVID-19 subjects revealed inflammation of the intestinal endothelial lining and mesenteric ischemia [34]. Whereas gastrointestinal symptoms have not been linked to a higher risk of death, they do appear to be linked to a longer disease duration [59,60].

Furthermore, increased levels of transaminases, overall bilirubin [41,43,51], and glutamyl transferase were found in multiple people with COVID-19 [56,61]. A series of COVID-19 patients showed modest lymphocyte infiltration, sinusoidal dilatation, uneven liver necrosis at autopsy [62,63]. Additional case series revealed Kupffer cell growth and persistent hepatic obstruction [64]. Cholangiocytes are vulnerable to direct virus-induced cytotoxicity because they express ACE2 [65]. However, it’s unclear if COVID-19’s liver impairment is caused by SARS-CoV-2 infection, inflammation, medication, or a combination of factors. The liver’s function should be examined on a regular basis since most medications under consideration as COVID-19 treatments are metabolized there.

### 4.3. Circulatory System

Increased D-dimer levels and extended prothrombin duration, and overt thrombotic symptoms, have been reported in a large percentage of COVID-19 patients [33,35,41,43,51]. The viral RNA is found in blood samples obtained from COVID-19 patients [43,58]. ACE2 is expressed in smooth muscles and endothelium lining the blood arteries and veins in many organs, SARS-CoV-2 may specifically attack these tissues, causing endothelial dysfunction [26,34,42]. Additionally, high synthesis of pro-inflammatory mediators results in an imbalance among pro- and anti-coagulant factors, as well as platelet aggregation [35,66]. Alongside NET generation, a rise in tissue factor V and VIII, thrombin, and fibrinogen levels resulting in a hypercoagulation state and an elevated risk of systemic macro- and micro-thrombosis [30,35,66,67]. Fibrous exudates and thrombi are observed in tissue specimens obtained from COVID-19 patients [5,42,62]. Severe COVID-19 has been linked to a higher risk of cardiovascular complications, including a higher rate of clinical heart disease symptoms (increased heart rate), increased levels of cardiac biomarker levels, and irregularities in EEG [41,43,68]. The majority of COVID-19 patients had cardiomegaly, right ventricular dilatation [31], and moderate fibrosis [62], according to postmortem findings. Since ACE2 is abundantly expressed in cardiomyocytes, the heart is vulnerable to SARS-CoV-2–mediated organ dysfunction [26,34]. Pericytes, however, produce even larger amounts of ACE2 compared to cardiomyocytes, and hence can become attacked by the virus, resulting in capillary endothelial dysfunction [9,52]. Heterozygosis with respect to ACE2 activity reduction is proved to be enough to enhance the chances of heart disease [69]. Although the significance of circulating soluble ACE2 is unknown, it is considerably elevated in case of cardiovascular dysfunction due to COVID-19 infection, suggesting that it might be employed as a biomarker [63,69,70]. Nevertheless, cytokine storm may cause direct myocardial damage when combined with a hypoxic condition caused by pulmonary failure. In COVID-19, initial acute myocardial damage is linked to a greater risk of deaths. COVID-19 may increase cardiac tissue damage and dysfunction, therefore already existing cardiovascular disorders are linked to a poorer prognosis [44,48,71]. It is advised that heart function and standard biomarkers be monitored.

### 4.4. Respiratory System

Since, ACE2 is present in numerous cell types along the respiratory tract, lung is without a doubt is the most sensitive and impacted by COVID-19 infection [58,72]. In chronic cases, acute pneumonia is the most prevalent and significant clinical symptom [43,46]. The virus may infiltrate through mucosal membranes of upper respiratory tract or immediately infect bronchial as well as alveolar epithelial cells in the lower respiratory tract since ACE2 is present in numerous cell types along the respiratory tract [58,72]. Fascinatingly, nasal ACE2 expression is decreased in children compared to adults which explains age-related changes of COVID-19 infection [73,74]. The viral RNA is found in the sputum of infected patients even before the onset of clinical symptoms [75], and even 2 weeks after remission [58,76]. In comparison to non-smokers and healthy patients, people with chronic obstructive pulmonary disease, current and former smokers, overexpress ACE2 in airway cells, which might underlie at least part of the elevated chances of severe infection in these people [74,77,78]. In both the SARS-CoV and SARS-CoV-2 contagion, ACE2 serves similar to a double-edged sword: it works as a receptor for virus entry into host cells and protects lung from damage by counterbalancing the vasoconstrictive and pro-fibrotic effects of Ang II on pulmonary vascular and epithelial cells [79,80]. Virus-ACE2 internalization [81] or cleavage and shedding of ACE2 [63,82] reduces its expression while enhancing Ang II buildup, synthesis of the receptors for TNF-α and IL-6, and stimulation of M1 macrophages (pro-inflammatory state) [16,82,83,84]. Moreover, the viral capsid proteins may combine with Smad3 to inhibit the death of infected host cells, inducing tissue fibrosis mediated by transforming growth factor (TGF)-β [85]. Hyperinflammation and fibrosis were reported in critical COVID19 patients where galectin-3 was proposed as potential biomarker [86]. Similarly, suppression of INFß while higher level of IL-1α and TGF-ß were also proposed as biomarker for COVID patients with acute lung fibrosis [87]. The self-renewing alveolar epithelial type II cells respond to high ACE2 amounts which can be repeatedly targeted for viral entry, leading to a cycle of tissue injury and repair resulting in the replacement of gas-exchange areas with fibrotic lesions. ACE2 is expressed in lung progenitor cells. Therefore, viral replication in them can perturb lung tissue healing [88]. A genome-wide associated study discovered novel host gene variables that may lead to the improvement of respiratory dysfunction induced by COVID-19. SNPs detected in six genes (SLC6A20, ZTFL1, CCR9, FYCO1, CXCR6, and XCR1) are thought discovered to be potential significance [39]. Additionally, the ABO blood type has been linked to SARS and COVID-19 vulnerability [27,39,89]. ARDS, SARS, and COVID-19 all exhibit lung histopathology findings that are very similar [5,62,63]. Elevated counts of neutrophils and mononuclear cells scattered alveolar damage with proteinaceous exudates, and epithelial cell hyperplasia are among the symptoms. In other situations, giant cells with multiple nuclei have been discovered in alveoli [5,62,63]. Despite of the usage of anticoagulation agents, an increase of deep venous thrombosis was observed in COVID-19 cases [33,90]; this condition may worsen further leading to the respiratory failure. Impairment of lungs caused by COVID-19 can be classified into three phenotypes based on chest computed tomography results: (1) focal, multiple, potently over perfused opacities; (2) non-homogeneously diffused atelectasis; and (3) moderate/severe ARDS, alveolar edema and reduced complain [52]. Since these traits are linked to various pathogenic processes and illness development, individualized mechanical ventilation systems should be used to facilitate an effective recovery for every individual.

### 4.5. Urogenital Tract

To maintain normal kidney function, fine balance between the activities of ACE2/Ang (1–7) as well as Ang II must be maintained. Since ACE2 is found in kidney cells, especially at the border of proximal cells, they are vulnerable towards SARS-CoV-2 invasion [26,91]. Proteinuria, hematuria, elevated serum creatinine along with blood urea nitrogen levels have all been seen in people with COVID-19 in laboratory testing [92,93,94]. On computed tomography, anomalies in the kidneys indicative of inflammation and edema were also seen [92]. The severe kidney damage in COVID-19 patients is linked to a much greater danger (approximately 5 times higher) of in-hospital death than those without acute kidney injury [41,44,51,92,94]. Dialysis is required in a significant proportion of COVID-19 patients with acute renal damage [93]. During autopsy, viral antigens are discovered in urine as well as tissue specimens from kidney, mainly the tubular epithelial cells and podocytes [60,95,96]; histopathological study showed tubular damage in addition to reduced brush border integrity, vacuolar degeneration as well as necrosis [96]. There has also been evidence of erythrocyte aggregation in capillary lumen without the involvement of fibrous debris [96]. Despite the possibility of direct viral invasion of kidney, inflammatory response and antiviral treatment might cause nephrotoxicity; hence, biomarkers for renal health as well as the balance of fluid and electrolyte should be continuously managed for the reduction in the extent of kidney damage. It is also observed that males with COVID-19 have a greater case fatality rate, regardless of age [97]. This may be due to the observation that testicular and vas deferens cells express substantially more ACE2 than ovaries [91,98,99], which might help explain disparities in disease severity and, as a result, mortality. Additionally, genes located on the sex-chromosome along with the sex hormones may decide the gender-specific control of immune responses in COVID-19 patients [100,101]. The X chromosome possess a large repertoire of immune system related genes, including ACE2. This functional mosaic may have an impact on innate and adaptive immune systems, as well as the severity of illness [101].

### 4.6. Hematological Disorders

The impacts of COVID-19 on blood were first reported from patients in China. However, the exact molecular pathway remains to be elucidated. One of the clinical manifestations of the COVID-19 thought to enhance the rate of mortalities is Venous thromboembolism (VTE). Autopsy samples from COVID-19 patients reveals the widespread occurrences of microthrombi not only in blood vessels associated with lungs but also with many other organs. These findings highlight the essentiality of thromboprophylaxis towards the reduction in the COVID-19 severity especially in ICU patients. In the patients who died of COVID-19, the levels of lymphopenia, coagulation abnormalities and thrombocytopenia are highly enhanced. 

The pulmonary VTE causes sudden increase in the arterial CO_2_ pressure causing the increase in dead space ventilation. A study conducted in the New York involving 3334 COVID-19 patients correlates the direct association between VTE and the increased mortality rate [102]. The COVID-19 patients also show hypercoagulation however bleeding was observed to be less common which makes it different from the Disseminated Intravascular Coagulation (DIC). In case of acute DIC cases, bleeding is predominant along with low levels of fibrinogen and platelets while in chronic cases, levels of fibrinogen are elevated. In case of COVID-19, a mildly decreased levels of platelets induces the state of hypercoagulation [103]. 

### 4.7. Systemic Health Complications Post COVID-19 Vaccination

**Figure 5:**
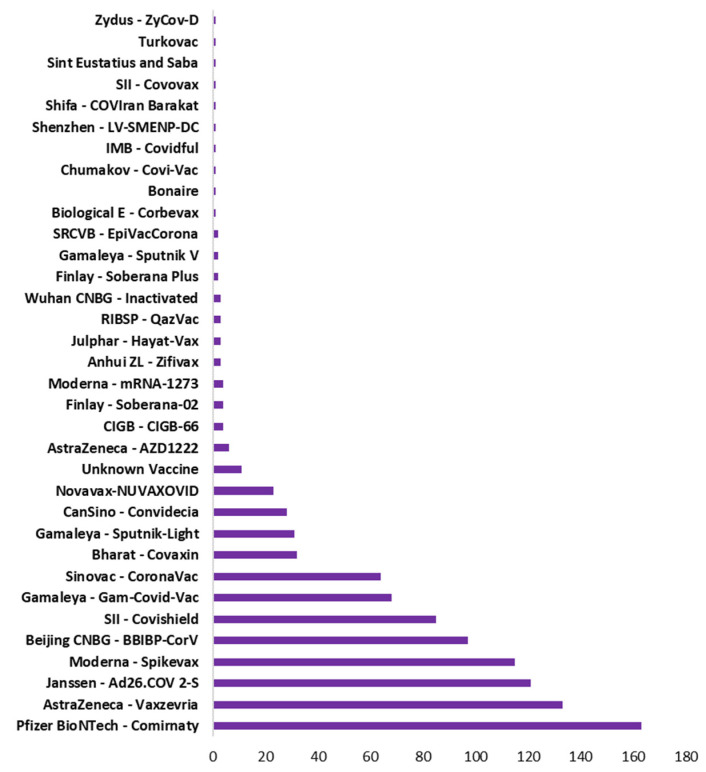
Type of vaccine with total number of countries used that vaccine.

**Figure 6:**
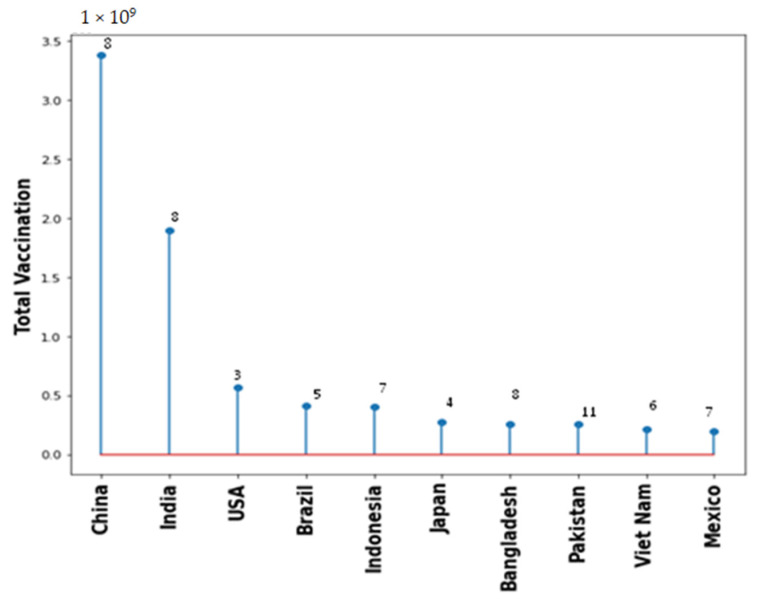
Total vaccination for the top 10 countries with number of types of different vaccine used.

Vaccination drive had been rapidly growing across all countries. Figure 5 shows the type of vaccine and number of countries used that vaccine. Pfizer BioNTech has been used maximally in 163 countries. However, AstraZeneca, Janssen and Moderna also used in 100+ countries. Figure 6 shows the top 10 countries based on total number vaccine dose on total population. China, India, and USA are the leaders in vaccination drive. India and China both used 8 different vaccines while USA used 3, as shown in Figure 6. The COVID-19 vaccines developed by the Pfizer and Moderna relies on the use of a combination of mRNA and nanoparticles for the efficient delivery of vaccines [104,105]. While, the vaccines developed by AstraZeneca, Gam-COVID-Vac and Johnson and Johnson relies on the recombinant DNA carried in a non-replicating adenovirus vector [106,107]. Both of these vaccine types produce the spike protein of SARS-CoV-2 against which neutralizing antibodies are produced within host body. The results obtained from the phase III clinical trials revealed that both the Pfizer as well as Moderna mRNA vaccines gave about 90–95% protection against COVID-19. Whereas the vaccines containing recombinant adenoviral vector exhibited a slightly lower degree of protection (70% and 91%, respectively). Both the vaccine types induce the generation of significant levels of neutralizing antibodies as well as the virus specific T cells detectable in the patient’s blood within 2–4 weeks of the administration [108,109,110]. These results were obtained from the clinical trials involving more than 100,000 volunteers from the global population. Although, the data obtained from the clinical trials ensures the safety of COVID-19 vaccines, numerous case studies have described serious complications with the involvement of multiple organs such as heart, brain and vascular system after vaccine administration [111,112,113,114,115,116,117,118,119,120].

### 4.8. Cardiac Complications 

Various case reports showed the induction of myocarditis post administration of COVID-19 mRNA vaccines. These are categorized by the Center for Disease Control and Prevention as acute myocarditis, confirmed myocarditis and probable myocarditis [120]. The data obtained from CDC confirmed the likelihood of myopericarditis in younger males which becomes predominant following the second mRNA vaccine dose compared to the first. Various theories have been proposed to understand the mechanism behind this association; (1) erratic activation of innate immune response by the mRNA of vaccine, (2) similarity between COVID-19 spike protein encoded by the mRNA vaccine and the cardiac proteins; (3) generation of hyperimmune response due to induction of high antibody titers, and (4) autoimmune response by the vaccine induced antibodies in patients with compromised cardiac health [121,122,123,124].

### 4.9. Myopathies

In COVID-19 patients severe damage and loss of skeletal muscles has been reported which may lead to a severe form of myopathy further complicating the disease outcome. Although, the mechanism underlying this condition is not revealed yet, it is believed that the virus induced cytokine storm causes the damage to muscle cells [125]. The condition of rhabdomyolysis which is a life threatening condition caused by muscle breakdown and death has been detected several days post the onset of COVID-19 symptoms [126]. This conditions correlates well with levels of the inflammatory markers [127]. 

Rhabdomyolysis has been previously observed as a rare case of complication due to vaccination in case of the influenza A H1N1 vaccine as well as a recombinant zoster vaccine [128]. Recently, severe case of rhabdomyolysis was observed in a young COVID-19 patient post administration of AstraZeneca COVID vaccine. Patient also had a deficiency of carnitine palmitoyltransferase II [129]. Another such case was observed in a male patient of 21 years having the asthma history who have received the first dose of Pfizer vaccine [130]. In a latest case, the patient administered with 2 doses of Pfizer mRNA vaccine developed anti-neutrophil cytoplasmic antibody associated vasculitis as well as the Pauci-Immune Crescentic Glomerulonephritis. However, the patient recovered post appropriate treatment [131].

### 4.10. Hematological Complications

In a few patients who received the recombinant adenoviral vector vaccine (chAdOx1-nCov-19) encoding for the viral spike protein developed the life-threatening conditions of thrombosis and thrombocytopenia. It was thought to be due to the induction of antibody response against the platelet factor 4 by the vaccine [132,133]. Other more common and less adverse side effects include headache, diarrhea, arthralgia, and fever which are generally self-limiting. 

In the year, 2021 a case study was reported of an old man (68 years) with an extremely severe attack of rhabdomyolysis 9-day post administration of first vaccine dose. This patient displayed multiple organ failure with damage to the liver, kidney, lung and bone marrow. The presence of elevated inflammatory markers was an indicative of the cytokine storm. The patient was administered with the immune-suppressive therapy targeted against multiple organs consisting of steroids, anakinra and eculizumab. Although the patient responded to the treatment indicated by the restored levels of the creatine phosphokinase but eventually he succumbed to the accelerated pace of deterioration and died after 48 days of vaccination [134]. 

Recently, administration of adenoviral vector COVID-19 vaccine has shown to induce coagulopathy in patients [135]. Additionally, two cases of cerebral venous sinus thrombosis were reported probably due to the autoantibodies against the platelet factor-4 indicating a probable immunological disorder [136].

Due to several recent reports, the development of a new syndrome following the administration of mRNA vaccine has been put forward termed as ‘Vaccine Induced Immune Thrombocytopenia’ [137,138,139,140,141]. A recent autopsy report from 58-year-old patient post 13 days of his first dose of viral vector vaccine indicated the presence of above-mentioned syndrome. The patient displayed multiple microthrombi in the heart, lung, kidney and choroid plexus [136]. The aggravation of immune thrombocytopenic purpura has also been reported post administration of mRNA vaccines. Scottish database shows the probable association between the viral vector vaccine and thrombocytopenic purpura [142,143]. 

### 4.11. Multisystem Inflammatory Syndrome (MIS)

The BNT162b2 mRNA vaccine produced by the Pfizer-BioNTech showed high efficiency with less side effects in clinical studies. In a case study reported in April 2021, a young male (20 year) displayed an early onset of nausea, non-bloody emesis, diffuse myalgias, abdominal pain on the day of vaccine administration. The next day, he showed signs of fatigue and pustules on the chest, face and back. After 48 h post vaccination, the patient exhibited hematuria as well as dysuria. The patient has no history of COVID-19 infection, was not on any medications or recreational drugs as well as no background of allergies. The patient received vaccinations in the childhood without any allergic responses. His two siblings also received both doses of BNT162b2 vaccines without any health complications [144]. Laboratory evaluations revealed the presence of systemic inflammatory response such as elevated levels of erythrocyte sedimentation rate, lactate dehydrogenase, C-reactive protein. Cytokine evaluation depicted high levels of IL-6, IL-10, IL-13. Blood examination revealed lymphopenia, enhanced granulocytes. The patient showed symptoms of systemic inflammation, acute hearing loss, pericardial effusion, progressive kidney injury, systemic capillary leak, multifocal ischemic strokes. 

### 4.12. Hematophagocytic Lymphohistiocytosis (HLH)

Another critical complication caused by COVID-19 vaccine is the hemophagocytic lymphohistiocytosis (HLH) observed in a 43-year-old farmer from China. HLH is a life threatening hyper-inflammatory condition induced due to the erratic activation of macrophages and T_C_ lymphocytes that can potentially lead to multi-organ failure. The patient developed vomiting, malaise and consistent high fever immediately post administration of the inactivated SARS-CoV-2 vaccine (first dose). The preliminary body examination indicated the presence of pancytopenia, increased levels of triglyceride, decreased fibrinogen levels, elevated levels of serum ferritin, low cytotoxicity levels of Natural Killer (NK) cells and the presence of the DNA from Epstein-Barr Virus (EBV) and bone marrow hemophagocytosis. In this patient, the immune response induced by COVID-19 vaccine caused the development of HLH with a background of the chronic EBV infection. The mentioned study highlights the importance of screening for the presence of chronic EBV or other viral infection before the vaccine administration [145].

### 4.13. Neurological Complications

Towards the end of 2020, several reports indicating the development of neurological complications post COVID-19 vaccination started emerging. The first report of two patients who developed transverse myelitis upon receiving viral vector vaccine. Till now, out of the 9442 adverse events post immunization with mRNA vaccines, 254 were showing neurological complications [114]. In Mexico, a nationwide study involving 704,003 patients who received first dose of BNT162b2 mRNA was undertaken to evaluate the adverse events. It was observed that from a total of 33 adverse events, 17 showed neurological complications such as seizures, Guillain-Barre Syndrome, exacerbation of lumbar radiculopathy, acute transverse myelitis. Whereas the proportion of non-serious neurological complications were about 600 from a total of 100,000 vaccine administered patients [146]. The most common side effects observed after COVID-19 vaccination are myalgia (60%) and headache (68%) [147].

## 5. Vaccination Impact on Long Term COVID

Long-term COVID-19 incidence is minimized by vaccination, which prevents people from being infected in the first instance. The injections may help guard against the disease by reducing the amount of time the virus has complete freedom in the body. Bar-Ilan University in Safed, Israel conducted a test on 3000 people and surveyed their long term COVID-19 condition that is the major indicator for the organ failure at the later stage. This study showed that vaccinated participants had 54% less chances of headaches, 64% less probability for fatigue and 68% less likely for the muscle pain [148].

## 6. Gender Based Difference of Vaccination

There is a limited amount of gender-specific studies of the efficacy and adverse effects of the COVID19 vaccination. Baden et al. reported that vaccine efficacy was similar on male and female [149] while Bignucolo et al. showed higher efficacy on male population compared to female population [150]. Despite the minimal risk of infection in the reproductive system, vaccination has been linked to pregnancy difficulties in various studies [151,152,153,154,155,156,157,158,159,160,161]. Omeish et al., 2021 presented a cross-sectional study that showed gender-related differences in adverse effects after vaccination [162]. In this study, there was a significant difference between male (85.8%) and female (92.3%) participants’ side effects following the first vaccine dosage, however this difference was not significant following the second dose. Johnson and Johnson vaccine also reported for its rare blood-clotting side effect that has affected predominantly female recipients. However, the published findings were insufficiently conclusive, suggesting the need for more gender-specific research on diverse geographic populations to determine the post-vaccination adverse effects on male and female populations. 

## 7. Vaccination and Multiorgan Failure: Case Studies 

### 7.1. CS-1

One fatal incidence of MIS-A (multi system inflammatory syndrome in adults) occurred after a complete COVID-19 vaccination in a patient who had a prior spontaneous SARS-CoV-2 infection detected 6 weeks before the beginning of MIS-A symptoms. Cases of MIS-A comprised three days of fever, laboratory evidence of inflammation, neurologic and mucocutaneous clinical symptoms, and severe cardiac disease that included systemic hypotension advancing to cardiogenic shock. Capillaritis and multiorgan microvascular thrombosis histopathologic findings in conjunction with clinical symptoms and laboratory results are consistent with MIS-A. On physical examination, significant blood loss may suggest a diffuse intravascular coagulation–type image in which diffuse micro thrombosis depleted platelets and clotting factors. Although significant cardiac impairment in the context of large fluid volumes and intraperitoneal hemorrhage may have led to multiorgan failure, an etiology of clinical deterioration was likely multifaceted [163].

### 7.2. CS-2

This study presents the first instance of HLH in a healthy person upon receiving COVID-19 vaccination. After first dose of the virus inactivated vaccine, a Chinese farmer (43 year) had lethargy, nausea, and a prolonged high fever. Pancytopenia, elevated lipid, and reduced fibrinogen were observed during the initial examination. Further testing revealed high blood levels of ferritin levels, decreased NK cell mediated cytotoxicity, and the presence of EBV DNA. Hemophagocytosis was detected in the bone marrow. As a result, HLH was verified. HLH is a potentially fatal hyperinflammatory illness characterized by abnormally activated macrophages and cytotoxic T lymphocytes that can lead to multiple organ failure. According to the findings, it is critical to rule out the existence of active or subclinical EBV infection (or other common viruses) before administering these vaccines [145].

### 7.3. CS-3

In this instance, a White lady (48 year) arrived at the emergency room with complaint of 3-day long malaise and stomach discomfort. A first assessment at another hospital revealed slight anemia and significant thrombocytopenia. The peripheral blood smear revealed a significant decrease in the count of platelets, with the occasional presence of schistocytes. Further studies revealed reduced fibrinogen levels in addition to the prolonged activated partial thromboplastin time and a significant increase in d-dimer, indicating disseminated intravascular coagulation. The viral RNA was not found using RT-PCR test from the nasopharyngeal swab sample. Following the report of newer headache onset, CT scan indicated the presence of cerebral venous sinus thrombosis. Even after the administration of unfractionated heparin, patient still had increasing levels of microthrombi with hemorrhagic stroke as observed using the magnetic resonance imaging (MRI) and magnetic resonance venography (MRV) of the brain. Another round of angiography revealed some new thrombi in hepatic and splenic veins. Furthermore, the investigation also revealed the administration of Ad26.COV2. S vaccine 14 days before the beginning of onset [164].

### 7.4. CS-4

On 19 April 2021, a healthy old man (68 year) with no risk factors received the first dose of adenoviral vaccines. He denied any further drug assumption. Since the patient was concerned about adverse effects associated to the surgery, blood tests were conducted 15 days before the vaccination, and it was confirmed to be normal. He was taken to the emergency hospital after 9 days due to dyspnea, acute malaise, severe stomach discomfort, myalgia, and difficulties ambulating. Even though the patient was receiving steroid therapy, his body temperature remained at 36.2 °C and did not display fever. During clinical assessment, patient had a mild clouding of the sensory system, as well as weakness in all four limbs but no significant localized neurological abnormalities. Tests performed within a few hours of symptoms onset showed increased serum levels of alanine aminotransferase, aspartate aminotransferase, creatine phosphokinase, creatinine, blood urea nitrogen, lactic acid. Inflammatory markers were high, and there was a quick and substantial rise in CK and myoglobin. The patient was moved to the critical care unit due to the fast advancement of multi-organ failure. Other typical causes of rhabdomyolysis were ruled out, including trauma, vigorous activity, recent surgery, alcohol use, toxins, autoimmune illnesses, and malignancies [134].

### 7.5. CS-5

This research examined clinical characteristics of 11 individuals in Europe (Austria and Germany) who developed thrombosis or thrombocytopenia after receiving adenoviral vector-based vaccine. To detect the presence of antibodies against platelet factor 4 (PF4)-heparin, ELISA was used, and for the detection of platelet-activating antibodies, a modified (PF4-enhanced) platelet-activation test was used. Here, 9 individuals showed cerebral venous thrombosis, 3 displayed splanchnic-vein thrombosis, 3 had pulmonary embolism, and 4 had various thromboses. Out of these 11 individuals, 6 patients died. In addition, 5 individuals were diagnosed with disseminated intravascular coagulation. Heparin was not administered to any patient before the onset of symptoms. All individuals who were positive for anti-PF4–heparin also showed the platelet activation. In 2 individuals, further tests with PF4–heparin antibodies revealed PF4-dependent platelet activation. This substantiates Immune thrombotic thrombocytopenia can occur in the uncommon case of ChAdOx1 nCov-19 vaccination [132].

## 8. Vaccinated and Non-Vaccinated

It is now widely accepted that vaccinated people are at lower risk compared to non-vaccinated individuals. A study at Bar-Ilan University, Israel suggested that dose of vaccine (Pfizer-BioNTech) produced high scale safety from long term COVID syndrome than the unvaccinated individuals. However, the study is not yet been peer reviewed [165]. Another study compared the health complications and several different parameters on vaccinated and unvaccinated individuals. This study showed that asymptomatic infection was found on 18.2% of the first dose vaccinated individuals while it was 12% to the corresponding unvaccinated population. In contrast to asymptomatic infection, hospitalization was higher on unvaccinated population by 6.8%. Similarly, more than five disease symptoms were reported in 22.2% of vaccinated population while this number went to 31.4% on unvaccinated population [166]. Another study from Israel, 5526 unvaccinated population data infected from COVID was collected and found that 4.6 per 100,000 were hospitalized while it was 0.3 per 100,000 individuals on vaccinated population. However, for vaccinated population only 596 cases were available. Similarly, the data of death cases was 0.6 per 100,000 on unvaccinated population and 0.1 per 100,000 on vaccinated cases [167].

A recent study conducted from June 2021 to January 2022 where 4,079,234 individuals (age group 12–17) were administered with mRNA vaccine. Multisystemic inflammatory syndrome (MIS-C) were reported in 9 cases that could directly relate with the multi organ dysfunction. Out of these 9 cases, 8 were male and 3 had evidence for past SARS-CoV2 infection. The reporting rate of MIS-C was 1.1 per million doses of vaccine while it was 113 on the same age group infected by SARS-CoV-2. This study is still not peer-reviewed but showed the prevalence of MIS-C cases following mRNA vaccine in children [168]. 

## 9. Conclusions

After the detection of the first case of COVID-19, several complications ranging from mild to severe associated with the viral pathogenesis have been reported. Many of these complications resulted in the multiple organ dysfunction and failure. Due to the emergency of developing an effective therapeutic regime, several researchers tried their hands on creating COVID-19 vaccines which included the use of viral mRNA, envelope glycoproteins, DNA etc. The rapidly increasing number of deaths or severe deformities in survived patients literally forced the healthcare systems across the globe to test the efficacy of these vaccine candidates in clinical trials. Following this, these vaccines were made available to the governments of various countries without testing for their long-term complications. This assurgency created explicit usage of vaccines in different age groups many of which showed serious complications leading to multiple organ failure. Thus, although COVID-19 vaccines have been instrumental in bringing down the rate of mortality and disease transmission, various moderate to serious complications continue to emerge from various parts of the globe eventually leading to multi organ failure. This situation demands the immediate attention and in-depth studies to evaluate the immediate and long-term complications of various vaccine types in people from different age group and also various approaches for the betterment of the existing vaccines. Case studies are reported where the major complications including multiorgan failure have emerged after the vaccination. However, there is no study to establish strong correlation between vaccination and multiorgan failure. The biggest case series was performed in Austria where 11 patients were reported for thrombosis or thrombocytopenia after vaccination leading to multiorgan failure. Multiple organs, including the heart, lungs, kidneys, lymphocytes, testes, nervous system, and liver, are reportedly equipped with ACE-2 receptors for SARS-CoV2. Consequently, SARS-CoV2 can infect these organs by adhering to ACE-2 receptor-bearing cells. Lung fibrosis, which was found to be prevalent in severe COVID patients, might indirectly impact the damage to other organs. However, direct correlation between the fibrosis and observation of SARS-CoV2 RNA is still debatable. Dormant form of viruses in these fibrous tissues are also not reported. This concludes, in SARS-CoV2 infection, fibrosis may indirectly cause damage to other organs via affecting oxygen supply, cytokine storm, dysregulated immunological responses, coagulation failure, and infiltration of inflammatory cells.

Overall, this review presents a detailed study on COVID-19 infection and its complications including multi organ failure in vaccinated and non-vaccinated individuals. Moreover, current available data is not sufficiently large to draw any strong conclusion on instituting any relation between vaccination and multiorgan failure due to small sample size and lack of evidence-based reports. Moreover, the biological and pathological reasons behind these major complications are still not completely understood and more future studies on this aspect would provide greater details. 

## Data Availability

Not applicable.
